# Improving access for patients – a practice manager questionnaire

**DOI:** 10.1186/1471-2296-7-37

**Published:** 2006-06-19

**Authors:** James G Meade, James S Brown

**Affiliations:** 1 Lakeside Medical Practice, Enniskillen, Co. Fermanagh, Northern Ireland; 2 Institute of Postgraduate Medicine, University of Ulster, Coleraine, Co. Londonderry, Northern Ireland

## Abstract

**Background:**

The administrative and professional consequences of access targets for general practices, as detailed in the new GMS contract, are unknown. This study researched the effect of implementing the access targets of the new GP contract on general practice appointment systems, and practice manager satisfaction in a UK primary health care setting.

**Methods:**

A four-part postal questionnaire was administered. The questionnaire was modified from previously validated questionnaires and the findings compared with data obtained from the Western Health and Social Services Board (WHSSB) in N Ireland. Practice managers from the 59 general practices in the WHSSB responded to the questionnaire.

**Results:**

There was a 94.9% response rate. Practice managers were generally satisfied with the introduction of access targets for patients. Some 57.1% of responding practices, most in deprived areas (Odds ratio 3.13 -95% CI 1.01 – 9.80, p = 0.0256) had modified their appointment systems. Less booking flexibility was reported among group practices (p = 0.006), urban practices (p < 0.001) and those with above average patient list sizes (p < 0.001). Receptionists had not received training in patient appointment management in a quarter of practices. Practices with smaller list sizes were more likely than larger ones to utilise nurses in seeing extra patients (p = 0.007) or to undertake triage procedures (p = 0.062).

**Conclusion:**

The findings demonstrated the ability of general practices within the WHSSB to adjust to a demanding component of the new GP contract. Issues relating to the flexibility of patient appointment booking systems, receptionists' training and the development of the primary care nursing role were highlighted by the study.

## Background

In a typical day in Northern Ireland, 30,000 people see a family doctor or practice nurse. Indeed Patient access has been made a major objective within the government's recent primary care strategic framework [1]. An **Access Target **(**AT**) was outlined for United Kingdom general practice, in the new general practitioner contract between practices and Health Boards [2] and linked to an enhanced service payment if achieved. This AT came into effect on the 1^st ^of April 2004 and stated that "by 2004, all patients will be able to see a primary care professional within 24 hours and a GP within 48 hours". Practices were required to provide evidence to the relevant Health Board [3] that they were meeting this standard, following which they would receive a payment. Payments for meeting this access standard have since been reviewed [4].

A wide range of appointment systems have been used to meet patient requests for consultations [[5-7], and [8]]. Practice managers are involved in patient access [9,10] and to date seem satisfied with the introduction of AT [10]. Nevertheless, the Royal College of General practitioners (RCGP) in a comprehensive review questioned the appropriateness of using this standard [11]. Indeed Prime Minister Tony Blair has been publicly questioned about the inflexible way some practices have tried to achieve the AT by allowing patients to only book an appointment up to 48 hours in advance [12]. A Healthcare Commission report also found that 12% of patients were unable to see a GP within two working days and 13% did not get an appointment within that time because they wanted to wait longer to see a particular GP or for an appointment at a more convenient time [13].

No previous research on the management of primary care patient appointment systems was identified in Northern Ireland. The aim of this study was to survey practice managers in the WHSSB area and to examine what effect, if any, the AT was having on the management of patient appointments, and on practice staff, by assessing practice manager satisfaction with the scheme. We also explored whether any practices limited advance booking of appointments and in what way as it might suggest less ease of access for patients.

## Methods

A postal questionnaire survey of the 59 general practices in the WHSSB area was carried out between the 14^th ^of August and 31^st ^of October 2005 (see Additional File 1). It examined staff roles in managing patient access, the management options based on previous research findings [7,8], and assessed how AT might be changing these options. It also looked at the flexibility in appointment booking offered and compared practice manager satisfaction [14] with the introduction of AT. The questions relating to satisfaction [14] were based on three different aspects of task-related satisfaction (resource adequacy, challenge and workload management) and one based upon general satisfaction. The short scale questionnaire was used in this setting and modified for practice managers. The questionnaire had been used in a range of previous quality employment surveys where the Cronbach alpha coefficient was 0.55. The Cronbach alpha coefficient in this study was 0.817 among these 7 questions. The questionnaire was first piloted among 15 general practices in a district council area outside the WHSSB area. Descriptive data about each practice was supplied by the WHSSB. The questionnaire, along with a brief covering letter was sent to the general practitioners in the practice, with the request that the practice manager complete and return the questionnaire. Non-responders at the end of 4 weeks were sent another questionnaire and letter by post. All data were statistically analysed using version 12 SPSS. Comparisons were made between different practice profiles and AT introduction using Chi square testing, independent t test sampling, odds ratios and confidence intervals as appropriate.

## Results

### Appointment systems

Managers from 56 (94.9%) practices completed questionnaires. Practices used a variety of appointment systems to manage patient access (see table 1). All of the practices in the area (n = 59) had applied for and received enhanced service access payments for meeting the AT. Some 52 practices (92.9%) kept computerised records of appointments, with four practices (7.1%) using paper records only.

**Table 1:** Types of appointment systems used by general practices in the WHSSB area

Appointment System	Practices	Percentage
Open surgeries (alone or 1 system used among others)	9/56	16.1%
Open surgeries (exclusive with no pre-booking at all)	2/56	3.6%
Some form of pre-booking	54/56	96.4%
Pre-booked	50/56	89.3%
Pre-booked with some emergency slots	46/56	82.1%
Pre-booked with some appointments reserved for 48 hours	33/56	58.9%
Telephone consultations	48/56	85.7%
Telephone triage	28/56	50.0%
Nurses involved in telephone triage	14/28	50.0%
Extras (Patients who needed to, or who requested to, be seen when routine appointment slots were all occupied)	47/56	83.9%
Extras given a specific time to be seen	33/47	70.2%
Extras advised to wait their turn	17/47	36.1%
Nurses involved in seeing extras	20/47	41.9%

Most practices n = 32 (57.1%) had changed the way that they managed requests from patients for consultations as a result of AT. The remaining 24 practices (42.9%) had not altered their appointment systems.

The n = 32 practices reported how different appointment systems had changed since the introduction of AT (see table 2). Practice managers rated how particular appointment systems had been affected in their own practice using a scale -1 to 1, where -1 = used less, 0 = no change and +1 = used more.

**Table 2:** How particular types of appointment systems were altered in those practices, which reported making changes to patient access management, since the introduction of access targets*

Appointment system in use or previously in use prior to access target introduction.	Number of practices affected	Mean score of change	Standard deviation of mean score	Skewness	Skewness std. error
Telephone Consultations	28/32	.36	.621	-.407	.441
Open Surgeries	5/32	.00	.707	.000	.913
Pre booked appointments	28/32	.21	.787	-.411	.441
Pre booked appointments with more emergency slots	28/32	.82	.390	-1.775	.441
Pre booked appointments with more slots held for 48 hour booking	17/32	.76	.434	-1.3272	.550
Extra patients	23/32	.35	.573	-.132	.481
Telephone triage	12/32	.17	.389	2.055	.637

*Practice managers were asked to rate change to individual appointment systems on a scale of -1 to 1.

-1 = appointment system used less or not used in the practice since AT introduction

0 = No change to how appointment system is used in the practice

+1 = Appointment system used more or now used in the practice since AT introduction

There had been an increase in all types of pre-booked appointment systems, particularly the use of "emergency slots". Many practices reported seeing more patients who had not pre-booked appointments; referred to as "extras". Practices also reported an increase in telephone consultations. There was no overall change in the use of open surgeries (i.e. with no pre-booking required) in the Board area.

More practices (n = 19/26) in socio-economically deprived areas made changes to their appointment systems, compared to practices (n = 13/28) in more socially advantaged areas. The change in appointments systems ratio was 3.13 (95% CI 1.01 – 9.80, p = 0.0256).

### Flexibility in booking appointments

Among the 54 practices which used appointment booking, 53 (98.1%) offered patients the option of booking appointments more than two days in advance. Despite this, a significant number of practices n = 32 (59.3%) restricted the time in advance that patients could make bookings (see table 3). This ranged from one to 15 weeks, with median and mean times each of four weeks. The other n = 22 (40.7%) did not restrict advance appointments.

**Table 3:** Practice profiles and limiting advance booking of appointments

Practice	Time limit exists for booking appointments in advance		
	No	Yes	Total

Mean Patient list size	3640.50	6842.78	
Single-handed	10	3	13
Group	12	29	41
Rural	14	4	18
Urban	8	28	36
Deprivation	11	15	26
No deprivation	11	17	28

Practices that limited the time allowed for patients to make advanced appointment bookings had larger list sizes (mean 6843, SD 2706) than those which did not limit advanced bookings (mean 3641, SD 2213), mean difference 3202 (1802 – 4602), p < 0.001.

Group practices were also more likely to limit how far in advance a patient was able to book an appointment when compared to single-handed practices. When single/group practices were cross-tabulated with practices which limited/did not limit advance booking, the chi square with Yates correction = 7.416 (p = 0.006).

In addition, urban practices were also much more likely to limit appointment booking when compared to rural-based practices. When urban and rural practices were cross-tabulated with practices which limited or did not limit advance booking, the chi square with Yates correction = 13.126 (p < 0.001).

After controlling for the confounding effects of rural/urban status, the odds of restricting appointment booking was 10.15 times more likely in group practices than it was in single-handed practices (95% CI = 3.86 -26.67, p < 0.001 (Mantal-Haenzel method)).

### Practice management, staff roles and access targets

Practice managers were generally satisfied with AT and the management of patient access within their practices (see table 4). The mean satisfaction score was 5.57 (SD = 4.28). Most practices n = 41 (74%) had receptionists who had received some training in appointment management and patient access. Nonetheless, 14 (25.5%) had not received any training. Only 3 (7.5%) practice managers reported that their receptionists had received training in appointment management or patient access outside the practice setting. The absence of training was not associated with different practice profiles or patient list size.

**Table 4:** Aspects of practice manager satisfaction relating to AT introduction

Aspects of Practice Manager satisfaction	Responders	Mean score *	Std. Deviation
Practices' approach	54	.91	.784
Perspective valued	55	.93	.690
Amount of work involved	55	.55	1.152
Information on AT	55	.84	.938
Understanding day to day goals	55	1.13	.610
Use of resources	55	.56	.977
General satisfaction – facet free	56	.77	.831

*Mean based on scale from -2 (Strongly disagree) to 2 (strongly agree).

Practices that used nurses to see "extras" had smaller list sizes (Mean = 4088, SD 2699) than those that did not use their nurses in this way (Mean = 6507, SD 2982), mean difference 2419 (693 – 4145), p = 0.007 (see table 5).

**Table 5:** The role of the Primary care Nurse and patient list size

Nurse role	Practices	Mean patient list size	Std. Deviation	Std. Error
Does not see extras	25/47	6507.44	2981.94	596.39
Sees extras	20/47	4088.35	2669.44	596.91
Does not do telephone triage	11/27	7001.55	2694.336	812.373
Telephone triage	14/27	4550.14	3384.315	904.496

Practices that used nurses to telephone triage patients requesting an appointment also had lower list sizes (Mean = 4550, SD 3384), and those that did not use them for triage (Mean = 7002, SD 2694), mean difference 2452 (-137 – 5040), p = 0.062 (see table 5).

## Discussion

### Summary of main findings

Practices used a variety of appointment systems and techniques to manage the patient access requirements of the new GMS contract (table 1). These systems have become increasingly structured since the introduction of AT and the associated financial gain. The odds of practices in deprived areas making changes to their appointments systems was three time greater than for those in more affluent areas. (Odds ratio 3.13 -95% CI 1.01 – 9.80, p = 0.0256). An increase in accommodating patients with earlier appointments was observed (table 2). This included more telephone consultations, triage, appointments within 48 hours and "extras" been seen. This may reflect a GP service more responsive to patient demand encouraged by the financial reward offered by the government within the new GP contract.

Nevertheless, practices' experience in the WHSSB area in managing access targets differed considerably. A significant minority had in fact not made any changes (n = 24) to their appointment systems. It may be that these practices had already been meeting the access standard before the new contract was introduced and saw no need to make changes.

Practices with larger patient list sizes (p < 0.001), or with predominantly urban patients (p < 0.001) and group practices (p = 0.006), were more likely to restrict the time bookings could be made in advance (table 3). Socio economic deprivation status was not related to appointment booking inflexibility.

Most Practice managers were satisfied with AT and the management of patient access within their practices (table 4). Despite receptionists playing a major role in the management of appointments, a number of practices n = 14 (25.5%) reported that their receptionists had received no training in doing this at all.

Nurses were more likely to be involved in seeing extra patients (p < 0.01) or in carrying out triage duties (p = 0.062) the smaller the patient list size (table 5). This was a counterintuitive finding.

### Strengths and the limitations of this study

The high response rate to this postal questionnaire based study could indicate that practices felt that the issue of patient AT was important. The absence of data from the three practices (5%) which did not reply is unlikely to have had a major biasing effect on the results.

As no previous research on appointment systems in the Board area was found, it was not possible to directly compare appointment systems prior to the introduction of the AT arrangements and this was a weakness in the methodology. In addition, any comparisons with appointment management systems in English primary care organisations with The WHSSB must allow for the fact that the WHSSB is likely to be a more rural region.

### Comparison with existing literature

We have highlighted the fact that patient access has remained flexible in the WHSSB area, and that 98.1% of practices allowed advanced booking of appointments beyond 48 hours. This contrasted with recent findings from the Health Commission [14]. The difference may be due to regional variation, or perhaps by practice managers biased reporting of flexibility, whether it existed or not given that under the terms of the new contract it is currently seen to be "desirable".

We have identified a lower use of the open access approach than previously reported [7]. By contrast, the use of pre-booked appointments was higher in this study (96.4%) than in others. Telephone triaging and the use of "extra" patients' surgeries (table 1), were also utilised more frequently and given more allotted time than has been previously reported [7]. Perhaps some of these changes could be attributed to the introduction of AT?

### Implications for future research or clinical practice

We feel this is the first study in Northern Ireland to examine the effects of implementing AT in general practices. We have highlighted that Practices in deprived areas have made more changes to their appointment systems than those in more affluent areas. This in turn may have implications for the allocation of investment and resources within the WHSSB.

Further training in managing access may be required in light of the new contract's access target requirements. The lack of training for some staff was highlighted in the study and may have clinical governance consequences for practices and for the Board, which has a policy of encouraging all practices to use standardised training programmes for its employees. Further research to explore the varying nursing roles in different practice profiles, suggested in this study, in relation to access may contribute to the debate on patient access and AT. Given their central role in patient access, any debate about AT arrangements [4,15] should consider the role of nursing in primary care.

Our study raises other questions about the clinical benefit of AT. Do the changes reported in appointment systems lead to an improvement in patient care? What effect does the requirement to offer AT to patients have on their behaviour and attitude to health, particularly self-care? Furthermore, has AT had an impact on secondary care as a result of earlier referrals, or a change in the workload at Hospital Accident and Emergency departments or even in the use of out hour's services?

## Conclusion

GPs in N. Ireland have been able to adjust to this government target by making changes to appointment systems and practice managers have been satisfied with its introduction. Factors relating to flexible appointment booking, receptionists' training and the development of the primary care nursing role were issues highlighted. Although this study has limitations, it is based in a standard primary care setting in N. Ireland and has a very high practice manager response rate. The data should contribute to the widening and continuing debate throughout the United Kingdom about the ease and speed of patient access to primary care, and the effect this might have on the rest of the NHS.

## Competing interests

Both authors are practicing GPs. The research paper followed on from a thesis prepared for MSc in primary care, Faculty of Life & Health Sciences, University of Ulster, N. Ireland.

## Ethics

The Office for Research Ethics Committee, Northern Ireland (OERCNI) independently ethically approved the study (REC reference number: 05/NIR02/66).

## Authors' contributions

JGM conceived of the study, participated in all aspects of the study including design and coordination, performed statistical analysis and drafted the manuscript.

JSB participated in the design and coordination of the study and helped draft the manuscript.

Both authors read and approved the final manuscript.

## Funding

The Western Health & Social Services Board, N. Ireland has approved the study for funding.

## Pre-publication history

The pre-publication history for this paper can be accessed here:



## Supplementary Material

Additional file 1 Click here for file

## References

[B1] Department of Health, Social Services and Public safety (2005). "Caring for people beyond tomorrow".

[B2] BMA and NHS Confederation (2003). General practice contract and explanatory notes – The GMS contract.

[B3] Western Health Social Services Board (2004). Specification for a Directed Enhanced Service (DES) Access to Primary care Service.

[B4] Med Economics UK database GP magazine London.

[B5] Jiwa M, Mathers N, Campbell M (2002). The effect of GP telephone triage on numbers seeking same-day appointments. British Journal of General Practice.

[B6] Kendrick T, Kerry S (1999). How many surgery appointments should be offered to avoid undesirable numbers of "extras"?. British Journal of General Practice.

[B7] Luthra M, Marshall M (2001). How do general practices manage requests from patients for "same day" appointments? A questionnaire survey. British Journal of General Practice.

[B8] McKinstry B, Walker J, Campbell C, Heaney D, Wyke S (2002). Telephone consultations to manage requests for same-day appointments: a randomised controlled trial in two practices. British Journal of General Practice.

[B9] Checkland K (2004). Management in general practice: the challenge of the new general medical services contract. British Journal of General Practice.

[B10] Ahluwalia S, Offredy M (2005). A qualitative study of the impact of the implementation of advanced access in primary healthcare on the working lives of general practice staff. BMC Family Practice.

[B11] The Future of Access to General Practice (2004). Practice Based Primary medical care – Informing the debate. Royal College of General Practice & NHS Alliance.

[B12] BBC Television, Question Time (2005). BBC 1 TV programme.

[B13] News Independent. http://news.independent.co.uk/uk/health_medical/article310934.ece.

[B14] Cook JD, Hepworth SJ, Wall TD, Warr PB (1981). The Experience of Work: A Compendium and Review of 249 Measures and their Use.

[B15] Dewsbury A (2005). "New Appointment rules anger GPs". Practice management Update.

