# Supplementary material for: Improving access for patients – a practice manager questionnaire
Source: BMC Fam Pract. 2006 Jun 19;7:37. doi: 10.1186/1471-2296-7-37 (PMC1570467; doi:10.1186/1471-2296-7-37)
Supplement: Additional file 1 [file 1471-2296-7-37-S1.pdf]

14 This last section relates to your job satisfaction in dealing with the enhanced service to access as a practice manager. Please think of your job since the implementation of the New GMS contract and circle your thoughts.

a My practice's approach to the access enhanced service makes sense to me.

Strongly Disagree	Disagree	Neutral
Agree	Strongly Agree	N/A (Not applicable)

b My perspective on how to manage access is valued and encouraged within the general practice.

Strongly Disagree	Disagree	Neutral
Agree	Strongly Agree	N/A (Not applicable)

c The amount of work I am expected to do in relation to access management is reasonable.

Strongly Disagree	Disagree	Neutral
Agree	Strongly Agree	N/A (Not applicable)

d I have adequate information available which enables me to do my job well in relation to this access-enhanced service

Strongly Disagree	Disagree	Neutral
Agree	Strongly Agree	N/A (Not applicable)

e I understand the day-to-day goals in relation to patient access and the enhanced service

Strongly Disagree	Disagree	Neutral
Agree	Strongly Agree	N/A (Not applicable)

f The practice management of access makes efficient use of resources in terms of time and cost to the practice

Strongly Disagree	Disagree	Neutral
Agree	Strongly Agree	N/A (Not applicable)

g Considering everything, how satisfied are you with your job in relation to access and the enhanced service?

Extremely dissatisfied	Dissatisfied	Neutral
Satisfied	Extremely satisfied	N/A (Not applicable)

This is the end of the questionnaire, thank you very much for completing and returning your answers.

Please make any comments in the space below.

65070 - design+print - acos design (the print factory design studio) - www.theprintfactory.com - t. 028 6632 6960

ROYAL COLLEGE OF GENERAL PRACTITIONERS

CUM SCIENTIA CARITAS

Northern Ireland Council

SURVEY ON ACCESS TO GENERAL PRACTICE
STUDY SUPPORTED BY RCGPNI

Dr. J. Meade

PRACTICE MANAGER QUESTIONNAIRE

This study looks at Access to general practice. The access service itself was summarised by the NHS plan "by 2004 all patients will be able to see a primary care professional care within 24 hours and a GP within 48 hours." The questionnaire should only take 10 minutes to complete.

Thank you for completing and returning in the prepaid envelope.

PLEASE CIRCLE YOUR ANSWERS.

1 What is your time commitment as practice manger (please circle)?

Full time Part time

If part time please specify ____________________ (e.g. ¾, ½, ¼, etc)

2 What age group are you in (Please circle)?

20-29 30-39 40-49 50-59 60-69

3 What sex are you (please circle)?

MALE FEMALE

4 Are the practice receptionists involved in allocating appointments with GPs and /or appointments with nurses in the practice (please circle)?

YES NO

If NO, please go to Question 6

5 Have your receptionist staff received specific training in relation to appointment management (please circle)?

YES NO

If NO, proceed to question 6

If YES,

Was this training within the practice (please circle)? YES NO

Was this training provided outside the practice (please circle)? YES NO

6 Please circle the main way in which practice appointments are recorded?

Paper record of appointments

Computerised appointment system

7 What types of GP surgeries are used in your practice (Please circle any systems that your practice uses. There may be more than one system in use)

Telephone Consultations	YES	NO
Open surgeries (No pre-booking required)	YES	NO
Pre-booked appointments	YES	NO
Pre-booked appointments with some emergency slots	YES	NO
Pre-booked Appointments with some slots allocated and released within 2 working days for emergency or urgent appointments	YES	NO
"Extra" patients are seen after pre-booked surgeries are finished (Commonly referred to as "extras")	YES	NO
A telephone triage system is used. This is operated by a Clinician (GP or nurse) to assess whether patients will be seen as an "extra" or as an emergency	YES	NO

8 Do requests for appointments ever result in interruptions to GP consultations (please circle)?

YES NO

9 (i) If your practice accepts pre-booked appointments, can patients make an appointment more than 2 working days in advance? YES NO NOT APPLICABLE

(ii) Is there a limit to how far in advance a patient can make an appointment?

YES NO

If YES, what is the limit (eg 1 week, 1 month, etc) .... (Please specify). If NO, go to Question 10.

10 If patients are seen after pre-booked surgeries are finished ("extras"), Please circle any groups who see these "extra" patients

If no extras are seen, please go to question 12

GP Principal	Assistant GP	Locum GP	Registrar GP
Practice Nurse	Treatment Room Nurse	Nurse Practitioner	

11 Please circle the answer to the following statements regarding your practice

All extras are given a specific time to be seen	YES	NO
All extras told to come at one specific time and wait their turn	YES	NO

12 If the practice uses a telephone triage system operated by a clinician (GP or nurse) to assess whether a patient needs to be seen as an "extra" or as an emergency. Please circle who does the triage.

If no system exists, please go Question 13.

GP Principal	Assistant GP	Locum GP	Registrar GP
Practice Nurse	Treatment Room Nurse	Nurse Practitioner	

13 Has your practice made any changes on how it manages requests for appointments as a result of the new enhanced service to access as described in the New GMS contract introduced in 1/4/04 (please circle)?

YES NO

If No, proceed to Question 14

If YES, please circle the effect of this change, if any on the following:

Telephone Consultations

MORE LESS NO DIFFERENCE NOT APPLICABLE

Open surgeries

MORE LESS NO DIFFERENCE NOT APPLICABLE

Pre-booked appointments

MORE LESS NO DIFFERENCE NOT APPLICABLE

Pre-booked appointments with <u>more</u> emergency slots

MORE LESS NO DIFFERENCE NOT APPLICABLE

Pre-booked appointments with some slots allocated and released on the day for extras (commonly referred to as "extras")

MORE LESS NO DIFFERENCE NOT APPLICABLE

Extra patients are seen after pre-booked surgeries are finished

MORE LESS NO DIFFERENCE NOT APPLICABLE

A telephone triage system has been introduced since 1/4/04

YES NO ALREADY EXISTED

If telephone triage already existed in the practice before 1/4/04, the practice has employed this system since then in the following way

MORE LESS NO DIFFERENCE NOT APPLICABLE

Telephone triage (whether new or already existing) has resulted in interruptions to GP surgery consultations since 1/4/04

MORE LESS NO DIFFERENCE NOT APPLICABLE
